# Quality analysis of discharge instruction among 602 hospitalized patients in China: a multicenter, cross-sectional study

**DOI:** 10.1186/s12913-020-05518-6

**Published:** 2020-07-11

**Authors:** Miao-miao Yang, Wei Liang, Hui Hua Zhao, Ying Zhang

**Affiliations:** Department of nursing, Zhongshan Hospital Fudan University, 180 Fenglin Road, Xuhui District, Shanghai, 200032 China

**Keywords:** Chronic disease, Discharge, Quality, Education

## Abstract

**Background:**

The aim of this study was to understand the quality of discharge guidance for patients with chronic diseases, to clarify the gap between patient needs and the content of discharge guidance, and to provide a reference for health education and clinical path management of patients with chronic diseases in the future.

**Methods:**

A total of 602 inpatients with stroke, coronary heart disease, cancer, chronic obstructive pulmonary disease and diabetes from the chronic disease-related departments of 7 tertiary general hospitals in China were selected by convenience sampling. Measures included a demographic questionnaire and the Quality of Discharged Teaching Scale(QDTS). Descriptive analysis ANOVA and paired t-test were completed by SPSS 22.0 software.

**Results:**

The overall average score of QDTS in this survey was 155.79 ± 23.29. The total score of QDTS in chronic obstructive pulmonary disease was lower than coronary heart disease (*P* < 0.001) and cancer (*P* = 0.02). While coronary heart disease was higher than stroke (*P* = 0.01) and diabetes (P = 0.01). And the scores of patients on discharge guidance skills and effects were higher than 8.50.

**Conclusions:**

The level of the patients’ perception of quality of discharge insrtuction is middle to high. Managers should understand the characteristics of various departments, give corresponding guidance and help, and clinical nurses should understand the characteristics of ward patients and pay more attention to individual guidance.

## Background

The prevalence of chronic diseases is high and the course of disease is long and protracted [1]. The rise in chronic illness is shifting the role of the nurse to that of facilitator in the patient’s self-management process. The increase in availability of personal disease monitoring tools such as hand-held, self-monitoring blood glucose systems [2], and a focus on patients as consumers of health care is moving expectations around management of chronic conditions away from physicians to the patients themselves. Studies have found that providing patients with personalized health information and reminders can improve compliance with guidelines, health status and patient satisfaction [3, 4].

However, the provision of discharge education is challenging. In a population-based patient satisfaction survey, 11% of patients reported that they had not received adequate information about their diagnosis and treatment plan at the time of discharge [5]. Nearly 1 in 5 patients experiences an adverse event during this transition, with a third of these being likely preventable [6]. Discharge guidance is a form of education and communication designed by nurses and other medical personnel to provide patients and/or caregivers with important information about medical care [7]. As an important part of holistic nursing, discharge guidance is the premise and guarantee for patients to continue to comply with the doctor and recover after discharge [8]. After discharge, the care responsibility of patients is transferred from medical service providers to patients and caregivers, so comprehensive discharge instructions are necessary to achieve the safely transition between hospital and home [9]. Therefore, the purpose of this study is to understand the quality of discharge guidance for patients with chronic diseases, to clarify the gap between patient needs and the content of discharge guidance, and to provide a reference for health education and clinical path management of patients with chronic diseases in the future.

## Methods

### Study design and sample

A total of 602 inpatients with stroke, coronary heart disease(CHD), cancer, chronic obstructive pulmonary disease(COPD) and diabetes from the chronic disease-related departments of 7 tertiary general hospitals in Shanghai were selected by convenience sampling between August and October 2018. The inclusion criteria for patients were: age ≥ 18 years, met the diagnostic criteria for chronic diseases, had certain literacy skills, participated voluntarily and gave signed informed consent. Patients with a poor physical condition, such as severe impairment of vision and/or hearing, or who were unable to complete the questionnaire, were excluded. The study was approved by the Hospital Ethics Committee.

## Instruments

### Demographic questionnaire

The questionnaire designed for this study included: patient gender, age, marital status, living arrangement, living environment, educational status, employment, income, medical diagnoses, disease course, length of stay, hospitalization frequency, knowledge of disease, and take medicine regularly. The questionnaire is included as additional Supplementary Material.

### Quality of discharged teaching scale (QDTS)

QDTS was developed by Weiss et al. [10] and has been widely used in spinal cord injury, stroke, and orthopedic patients [11, 12]. Translation of the QTDS into the Chinese version was done with the process of translation, back-translation, culture adaption, expert consultation and pre-test, and then chose 167 coronary heart disease from a tertiary general hospital in Wuhan to test the reliability and validity of the Chinese version scale [13]. The Cronbach’s alpha of the whole scale was 0.92, and those for the subscales ranged from 0.88–0.94. The S-CVI/Ave value was 0.98 and all of the computed I-CVI values were greater than 0.80. The scale consisted of 24 items using a 0–10-point Likert scale in 3 sub-scales: teaching contents that patients thought they needed (6 items), teaching contents that patients received (6 items), and teaching skills and effectiveness (12 items). The total scale score is calculated by adding the content needed and the content received subscale scores, with higher scores indicating better quality. The total scale can be considered both a measure of receiver characteristics of the nursing process and the outcome of the nursing process of discharge teaching. The scale has been authorized by the author.

## Data collection procedures

Trained study research assistants completed informed consent procedures and arranged for completion of the sociodemographic information and QDTS on the day of patient discharge. All questionnaires were collected on site by the research assistants after completion. So, there were no missing items from the study. A total of 620 questionnaires were sent out and 602 valid questionnaires were analysed.

## Data analysis

Continuous variables were reported as mean standard ± deviation (SD). While categorical variables were expressed as frequency, percentage. ANOVA and paired t-test were used for comparison between groups. All data analyses were performed by SPSS 22.0 statistical software.

## Results

### Demographic and clinical characteristics of the participants

Of all the 602 patients, 62.1% were male and 37.9% were female with ranging ages from 18 to 95 years (mean: 62.21 ± 14.37 years). The average length of hospital stay was 11.55 ± 16.23 days. Participants’ demographic characteristics are shown in Table 1.

**Table 1:** Characteristics of participants (*n* = 602), China, 2018

	Characteristics	Mean (SD)	Frequency(n)	Percentage(%)
Gender	Male		374	62.1
	Female		228	37.9
Age	<60		221	36.7
	≥60		381	63.3
Marital status	Single		27	4.5
	Married		522	86.7
	Divorced/widowed		53	8.8
Living arrangement	Living alone		34	5.6
	Living with family		568	94.4
Living environment	Urban area		404	67.1
	Town		129	21.4
	Rural		69	11.5
Education level	Junior high school or less or less		231	38.4
	High school		233	38.7
	College or higher		138	22.9
Employment	employed		146	24.3
	unemployed		456	75.7
Household income per capita	≤2420		59	9.8
	2421 ~ 4000		157	26.1
	4001 ~ 5000		200	33.2
	≥5001		186	30.9
Payment methods	Public expense		535	88.9
	At own expense		67	11.1
Medical diagnoses	Stroke		131	21.8
	CAD		132	21.9
	Cancer		145	24.1
	COPD		88	14.6
	Diabetes		106	17.6
Disease course		4.21 (6.38)		
Length of stay		11.55 (16.23)		
Hospitalization frequency	First hospitalization		236	39.2
	Re-hospitalization		169	28.1
	Multiple hospitalizations		197	32.7
Knowledge of disease	Know-nothing		49	8.1
	Know part of it		281	46.7
	Master		272	45.2
Need to take medicine regularly for a long time	YES		513	85.2
	NO		89	14.8

### The quality of discharge teaching perceived by chronic disease patients

The overall average score of QDTS in this survey was 155.79 ± 23.29. And the total score of QDTS and the scores of each dimension of patients with different kinds of diseases are compared in Table 2. Further comparison of the total score of QDTS by pairwise comparison shows that COPD was lower than CHD (*P* < 0.001) and cancer (*P* = 0.02). While CHD was higher than stroke (*P* = 0.01) and diabetes (*P* = 0.01). There were no statistically significant differences between COPD and stroke (*P* = 0.10), COPD and diabetes (*P* = 0.10), stroke and cancer (*P* = 0.41), CHD and cancer (*P* = 0.10).

**Table 2:** Comparison of the total score and each dimension score of QDTS in discharged patients with different diseases (n = 602)

Item	Frequency(n)	QDTS – Needed Mean (SD)	QDTS – Received Mean (SD)	Teaching skills and effectiveness Mean (SD)	Total score Mean (SD)
Stroke	131	46.59 (12.55)	48.69 (9.85)	105.76 (15.29)	154.46 (22.08)
CHD	132	49.52 (11.33)	52.72 (9.11)	108.88 (16.99)	161.60 (24.70)
Cancer	145	47.48 (13.45)	49.56 (11.07)	49.56 (11.07)	156.75 (24.26)
COPD	88	47.65 (14.18)	45.69 (11.92)	45.69 (11.92)	149.23 (23.80)
Diabetes	106	44.62 (12.96)	47.74 (11.92)	47.74 (10.67)	154.33 (19.47)
F		2.234	6.834	1.821	4.159
*P*		0.06	< 0.001	0.12	0.002

*Abbreviations*: *CHD* coronary heart disease, *COPD* chronic obstructive pulmonary disease, *QDTS* quality of discharged teaching scale, *SD* standard deviation, *p*, *p*-value

Table 3 compares the total score of QDTS and the scores of each dimension of patients from different hospitals. The scores of QDTS – Needed were not significantly different between the 7 hospitals. However, we found statistically significant difference among analyzed hospitals according to QDTS – Received, Teaching skills and effectiveness, and the total score of QDTS.

**Table 3:** Comparison of the total score and each dimension score of QDTS in discharged patients with different hospitals (*n* = 602)

Item	Frequency(n)	QDTS – Needed Mean (SD)	QDTS – Received Mean (SD)	Teaching skills and effectiveness Mean (SD)	Total score Mean (SD)
A Hospital	122	45.30 (15.79)	48.45 (11.72)	109.20 (11.54)	157.65 (20.39)
B Hospital	83	49.08 (13.21)	50.16 (9.11)	103.19 (17.39)	153.35 (24.93)
C Hospital	85	48.06 (12.99)	46.95 (11.34)	103.68 (15.05)	150.64 (24.37)
D Hospital	98	49.52 (8.90)	52.01 (7.43)	109.61 (11.98)	161.62 (18.47)
E Hospital	78	45.14 (13.79)	46.59 (12.82)	107.19 (13.67)	153.78 (22.81)
F Hospital	56	46.16 (11.82)	47.43 (11.91)	96.55 (21.96)	143.98 (31.57)
G Hospital	80	47.53 (11.53)	51.91 (8.54)	112.13 (11.81)	164.04 (18.40)
F		1.726	3.983	9.082	6.508
*P*		0.11	0.001	0.000	0.000

*Abbreviations*: *QDTS* quality of discharged teaching scale, *SD* standard deviation, *p* p-value

### Comparison of content-needed and content-received score of discharged patients with different kinds of diseases (Table 4)

The results of Table 4 show that there are differences in the demand for guidance content among patients with different diseases. Patients with stroke, CHD and cancer have the highest demand for “medical processing information”. COPD patients have the highest demand for “seeking help information”. Diabetics have the highest demand for “Family members’ informational needs”. And the paired t-test showed that except for the two items of “ medical information “ and “treatment practice” of COPD patients were not satisfied, that is, the score of the content-received was lower than that of the content-needed. There was no statistically significant difference between the scores of other items needed and received.

**Table 4:** Comparison of content-needed and content-received score (n = 602)

Item	Stroke(*n* = 131)	CHD(*n* = 132)	Cancer(*n* = 145)	COPD(*n* = 88)	DM(*n* = 106)
	Mean (SD)	Mean (SD)	Mean (SD)	Mean (SD)	Mean (SD)
Information needed about taking care of oneself	7.64 (2.22)	7.87 (2.64)	7.82 (2.57)	7.89 (2.77)	7.27 (2.66)
Information received about taking care of oneself	8.17 (1.83)	8.77 (1.84)	8.21 (2.06)	7.93 (2.14)	8.06 (2.03)
*t*	−3.351	−4.314	−2.091	−0.240	−3.250
*P*	0.001	< 0.001	0.04	0.81	0.002
Emotion regulation information needed	7.56 (2.34)	7.9 (2.43)	7.74 (2.61)	7.68 (2.85)	7.25 (2.63)
Emotion regulation information received	8.19 (1.86)	8.79 (1.78)	8.32 (1.97)	7.53 (2.36)	8.00 (1.88)
*t*	−3.823	−4.536	−3.119	0.615	−3.389
*P*	< 0.001	< 0.001	0.002	0.54	0.001
Information needed about medical needs and/or treatments	7.92 (2.18)	8.48 (2.03)	8.08 (2.33)	8.02 (2.23)	7.31 (2.60)
Information received about medical needs and/or treatments	8.02 (1.85)	8.81 (1.54)	8.33 (1.95)	7.56 (2.17)	7.95 (2.09)
*t*	−0.553	−1.995	−1.509	2.211	−2.890
*P*	0.58	0.05	0.13	0.03	0.005
Practice needed with treatments and/or medications	7.68 (2.31)	8.34 (2.06)	7.77 (2.53)	7.94 (2.51)	7.14 (2.62)
Practice received with treatments and/or medications	7.98 (1.99)	8.67 (1.82)	8.10 (2.11)	7.32 (2.34)	7.64 (2.30)
*t*	−1.879	−2.355	− 1.853	2.856	−2.252
*P*	0.06	0.02	0.07	0.005	0.03
Information needed about who and when to call for problems or emergencies	7.91 (2.18)	8.48 (1.91)	8.01 (2.41)	8.07 (2.48)	7.77 (2.41)
Information received about who and when to call for problems or emergencies	8.13 (1.71)	8.87 (1.55)	8.24 (2.14)	7.82 (2.13)	8.08 (1.95)
*t*	−1.489	−2.611	−1.298	1.110	−1.600
*P*	0.14	0.01	0.20	0.27	0.11
Family members’ informational needs	7.87 (2.32)	8.40 (2.15)	8.05 (2.33)	8.05 (2.42)	7.87 (2.46)
Information received by family members	8.21 (1.72)	8.82 (1.54)	8.37 (2.10)	7.53 (2.45)	8.01 (2.10)
*t*	−1.850	−2.516	−2.064	2.272	−0.614
*P*	0.07	0.01	0.04	0.03	0.54
Content needed	46.59 (12.55)	49.52 (11.33)	47.48 (13.45)	47.65 (14.18)	44.62 (13.30)
Content received	48.69 (9.85)	52.72 (9.11)	49.56 (11.07)	45.69 (11.92)	47.74 (10.67)
*t*	−2.772	4.168	2.513	1.867	2.911
*P*	0.006	< 0.001	0.01	0.07	0.004

*Abbreviations*: *CHD* coronary heart disease, *COPD* chronic obstructive pulmonary disease, *DM* diabetes, *SD* standard deviation, *t* t-test pairs, *p* p-value

### Patients’ evaluation of discharge guidance skills and effectiveness (Table 5)

The average score of discharge guidance skills and effects are 8.53–9.03. The items with the highest and lowest scores are “ Do you like the way nurses guide”, and “ Can the information provided by the nurse address your concerns and questions “. The scores of other entries are shown in Table 5.

**Table 5:** Scores of patients on the evaluation of teaching skills and effectiveness ($\overline{x}\pm s$)

Item	Average score of each item
Do you like the way nurses guide?	9.03 ± 1.38
Do you get the same information from nurses, doctors, and other health workers?	9.02 ± 1.38
Can you understand the way the nurse instructs you to take care of yourself?	9.02 ± 1.39
Is it the right time for the nurse to provide you with self-care information?	8.96 ± 1.41
Does the nurse respect your religious beliefs or values?	8.95 ± 1.67
Will the nurse choose the time when your family or other caregivers can be present to provide you with information?	8.95 ± 1.49
Can nurses help you improve your confidence in your ability to take care of yourself at home?	8.94 ± 1.55
Can nurses listen to your concerns?	8.93 ± 1.53
Will the nurse check to make sure you understand the information she provides or masters her demonstration?	8.86 ± 1.67
Can the self-care information provided by nurses reduce your anxiety about returning home?	8.79 ± 1.74
How confident are you that you know what to do in an emergency?	8.65 ± 1.65
Can the information provided by the nurse address your concerns and questions?	8.53 ± 1.78

## Discussion

### The quality of discharge teaching is in the upper-middle level, and there are differences among different departments

The overall score of chronic disease patients’ QDTS in this survey was slightly higher than that reported by Weiss et al. [10]. It shows that the QDTS of chronic disease patients is at a high level, which may be because that the data of this study came from the large tertiary general hospitals, the nurses have higher education background, and the professional training in the hospital was more frequent and strict, so the nurses had higher professional literacy and could guide the patients better. As can be seen from Table 2 that the differences in the total score and the scores of the QDTS - received by different departments are statistically significant (*P* < 0.01). And further pairwise comparison found that the total score of QDTS of COPD was the lowest, which was lower than that of stroke, CHD, cancer, and diabetes. It could be caused by the following reason. COPD was an age-related condition, and several evidences suggested a relationship with a rapidly increasing aging [14, 15]. It seems like older patients are more likely to develop subtle cognitive deficits [16]. These deficits are often not recognized by clinicians and may impair understanding [17, 18].

In addition, Table 2 also shows that patients with CHD need the highest content dimension score, suggesting that even at higher levels of patient demand, the discharge guidance provided by the nurse can still meet the patient’s needs. It is recommended that the nursing manager further study the characteristics of the nurses and ward in each department. For departments whose guidance quality needs to be improved, managers should enhance their nurses’ understanding of discharge guidance, encourage them to learn from the experience of excellent departments, and guide and help the nurses actively explore the characteristics of patients and possible out-of-hospital needs, and constantly improve the quality of care. Recommendations for improving discharge teaching emphasize a patient and family-centered approach, in which content and teaching method should be tailored to patient/family characteristics and circumstances, rather than the typical approach of patient diagnosis with standardized information [19].

### The needs of discharge guidance for patients with different kinds of diseases can be met

The differences in the demand for guidance content among patients with different diseases may be related to the patient group and treatment characteristics of the department. After treatment, there may be surgical incision dressing change, rehabilitation and other medical treatment in patients with stroke, CHD, and cancer. Patients with COPD who encounter problems in acute exacerbation are more likely to seek medical help directly. The treatment of patients with diabetes needs to change their previous lifestyle and eating habits and persist in taking medicine, which requires the cooperation and help of their caregivers. It is suggested that the content of discharge guidance provided to patients should be emphasized according to the disease characteristics of the department.

### Chronic disease patients have a high evaluation of discharge guidance skills and effects, but there is still room for improvement

The average score of discharge guidance skills and effects are 8.53–9.03, indicating that chronic disease patients have a high evaluation of nurses’ guidance skills. However, the effect of discharge guidance still needs to be improved. Patients have the lowest score on “ Can the information provided by the nurse address your concerns and questions “. We suspect that the following factors contributed. First, inadequate coordination within multidisciplinary discharge teams. Both physicians and nurses overestimate patients’ knowledge, which may make them less likely to provide sufficient education [20, 21]. Second, lack of feedback. Patients may have difficulty in understanding these instructions. A study on quality of discharge practices and patient understanding found that among the patients who felt very familiar with the discharge guidance, 40% could not accurately describe the reason for hospitalization, and 54% were unable to recall the instructions for the scheduled follow-up [22]. Third, lack of standardization in discharge procedures. Although guidelines for discharge care currently endorsed by the National Quality Forum [23] and others [24, 25] provide excellent templates, research has shown that the implementation of these standards at the hospital and physician level is limited [26].

Therefore, it is the key to exploring the methods to improve discharge practices. A safe and patient-centered passage from the hospital should therefore include high-quality provision of discharge instruction [27]. For example, leaflets or brochures should be provided to the patients at discharge, including guidance and educational materials appropriate to the patient’s language and education level. Emphasis should be on the use of simple terms and shorter sentences [28]. In addition, these documents should focus on conveying key messages to patients, and focus on the information that patients need to know for self-management after discharge. Both written and oral discharge information should be communicated to the patient / family caregiver, focusing on assessment and ensuring that they understand the discharge information. Another method to improve comprehension is using a “teach-back” technique, which has been widely advocated as a teaching method to improve patient understanding of discharge instructions [29, 30]. So that health care providers can verify the patients have understood the information or correct misperceptions [31]. It is also suggested that discharge guidance should be divided into stages and health guidance should be integrated into daily nursing work. Besides, in the process of discharge guidance, attention should be paid to detailed guidance, so that patients can acquire more clear and understandable knowledge, reduce anxiety, and promote their competence in-home care. However, education alone cannot improve the quality of teaching. Leadership support to monitor discharge teaching quality, modify clinical care delivery systems as needed, and assess staffing patterns is critical to addressing this important but often overlooked aspect of care. Highlighting improved quality of pre-discharge teaching will support the strategic efforts of hospitals and caregivers to improve discharge care and outcomes [32].

## Limitations and recommendations

A limitation of this study was that all of the subjects in this study were from tertiary hospitals. Accordingly, the results of this study may not be applicable to other populations. Subsequent studies can be conducted in different regions and different capacities to further understand the quality of discharge guidance for patients with chronic diseases. Also focusing on interventions to improve patient empowerment will be important in the future.

## Conclusion

The results of this survey suggest that there are differences in the quality of discharge guidance in different departments, and the needs of patients with different kinds of diseases are different. Therefore, it is suggested that nursing managers should understand the characteristics of various departments, give corresponding guidance and help, and clinical nurses should understand the characteristics of ward patients and pay more attention to individual guidance. At the same time, we should actively explore how to provide more comprehensive and effective education in a short and limited time, further improve the quality of discharge guidance, and help patients meet the health challenges after discharge.

## Supplementary information

**Additional file 1.** Demographic questionnaire.

## Data Availability

The datasets used and/or analysed during the current study are available from the corresponding author on reasonable request.

## Abbreviations

ANOVA: Analysis of Variance
COPD: Chronic Obstructive Pulmonary Disease
CHD: Coronary Heart Disease
DM: Diabetes
QDTS: Quality of Discharged Teaching Scale
I-CVI: Item-Content validity index
S-CVI: Scale-Content validity index
SD: Standard Deviation
