# Supplementary material for: Quality analysis of discharge instruction among 602 hospitalized patients in China: a multicenter, cross-sectional study
Source: BMC Health Serv Res. 2020 Jul 11;20:647. doi: 10.1186/s12913-020-05518-6 (PMC7353724; doi:10.1186/s12913-020-05518-6)
Supplement: Supplementary file 1 — Additional file 1. Demographic questionnaire. [file 12913_2020_5518_MOESM1_ESM.docx]

ID**：___________ No __________**

**Demographic questionnaire**

| **Part A ：Basic information** |
| --- |
| 1. Age：_______ |
| 2. Gender：□Male □Female |
| 3. Marital status：□Single □Married □Divorced/widowed |
| 4. Living arrangement：□Living alone □Living with family |
| 5. Education level：□Junior high school or less □High school □College or higher |
| 6. Employment：□employed □unemployed |
| 7. Household income per capita： □≤2420￥ □2421～4000￥ □4001～5000￥ □≥5001￥ |
| 8. Payment methods： □Public expense □At own expense |
| 9. Living environment： □Urban area □Town □Rural |
| **Part B：Data related to chronic diseases** |
| 10. Type of diseases ：  □Stroke □Coronary Heart Disease □Cancer □Chronic Obstructive Pulmonary Disease □Diabetes |
| 11. Disease course：_______years |
| 12. Length of stay：_______days |
| 13. Hospitalization frequency：  □ First hospitalization □ Re-hospitalization □ Multiple hospitalizations |
| 14. Knowledge of disease：□Know-nothing □Know part of it □Master |
| 15. Need to take medicine regularly for a long time：  □YES □NO |
